# Supplementary material for: Are carbon emissions trading and green financial instruments synergistic? -Comprehensive quantitative research based on content analysis
Source: PLoS One. 2024 Mar 7;19(3):e0298601. doi: 10.1371/journal.pone.0298601 (PMC10919601; doi:10.1371/journal.pone.0298601)
Supplement: S1 Data — (ZIP) [file pone.0298601.s001.zip › Data/地级市Z_F工作报告.zip!/╔╜╬≈/┤≤═1⁄4╩╨/└┤╫╘╣1⁄2╓┌║┼í╛┬φ┐╦╩2╛▌═°í┐_2015.docx]

各位代表:

现在,我代表市人民政府向大会报告工作,请予审议,并请市政协委员和其他列席人员提出意见。

一、一年来工作回顾

2014年是不寻常的一年。面对复杂严峻的形势,全市上下深入贯彻落实党的十八大、十八届三中、四中全会和习近平总书记系列重要讲话精神,在省委、省政府和市委的正确领导下,攻坚克难,奋力拼搏,十大工程扎实推进,四城联创有序开展,争先进位取得成效,全省年度目标任务考核排名由第五位进至第三位,综合排名由第七位进至第五位,各项工作稳中有为,经济社会发展迈上新台阶。

(一)经济发展稳中有进。地区生产总值首次突破千亿,同比增长7.4%,全省第一;规模以上工业增加值增长10.3%,全省第一;公共财政预算收入首次突破百亿,增长11.0%,总量由全省第八位进至第六位;社会消费品零售总额增长11.6%;固定资产投资增长3.5%;城镇、农村居民人均可支配收入分别增长8.1%、10.8%。城乡居民收入增速快于GDP增速,农村居民收入增速快于城镇居民收入增速。粮食产量20.8亿斤,实现“五连增”。9项安全环保约束性指标全面完成。实体经济健康发展,出台落实一系列稳增长政策措施,实行“同煤同用”“同机同用”“同材同用”,加强政银企对接,开展点对点帮扶,银行存贷比同比提高3.2个百分点,落实煤炭行业三项政策、减轻企业负担34.69亿元,同煤、国电、威奇达等大企业对全市工业经济增长的贡献率达到105.2%,新增规模以上工业企业18户,新增小微企业2101户。大项目建设明显加快,深入开展“项目见效年”活动,“六位一体”推进重点工程,大张高铁环评获批、开工在即,煤制气项目环评通过技术评估,同煤塔山电厂二期等3个低热值煤发电项目开工建设,全国首个采煤沉陷区国家先进技术光伏示范基地获批启动,进口肉类口岸查验场获国家批复、园区道路已经开工。

(二)改革开放不断深化。制定实施转型综改“2255”行动计划。深化资源要素、体制机制、民生领域等十项重点改革。政府出资1000万元设立科技投融资平台,由晋商银行放大10倍扶持中小企业科技成果转化,已向4户企业放贷2000万元。设立1000万元人才专项资金。“千人计划”专家人才引进实现零的突破,晋投玄武岩院士工作站今天挂牌成立。新登记市场主体增长26.89%。迁入城镇常住人口增长339%。左云经济技术开发区申报通过省专家评审。三医院、五医院、同煤总院分别牵头成立医疗联合体,一医院、三二二医院成功升级三甲。阳光小额贷款公司成为华北地区首家新三板挂牌的小额贷款公司,绿苑饮品、上皇庄岗酒、吧吡啦食品、纠偏古膳要道食品在上海Q板挂牌。市商业银行升级更名为大同银行,并在北京等地设立分支机构7处,京都村镇银行成立。航空旅客吞吐量增长13.99%,全省第一。外贸出口增长40.7%,全省第一。蒙晋冀毗邻地区(乌兰察布-大同-张家口)合作区建设纳入京津冀协同发展规划,上升为国家战略。

(三)产业转型步伐加快。大力推进百园立农工程,新建各类农业园区33个、标准化养殖园区169个,新增设施农业3.3万亩。大力推进百企强市工程,不断延伸煤炭产业链条,新增火电装机33万千瓦。被国家能源局确定为新能源示范城市,新增风电装机30万千瓦、光伏发电装机14万千瓦。同煤60万吨甲醇项目试生产、10万吨活性炭项目投产,同车10万吨活性炭项目一期基本建成、和谐30吨轴重大功率交流传动电力机车综合型试验完成,大秦公司3万吨重载列车试验成功。被商务部等十部委确定为全国66个区域流通节点城市之一。万昌物流园区进入全国物流园区50强。设立中国摄影展览馆大同中心。成功举办云冈文化旅游节、全国物流园区年会、国际汽车文化节等活动。跻身中国宜商城市竞争力第65名、旅游城市吸引力第41名、避暑旅游城市第11名。旅游总收入增长19.15%。三次产业结构调整为5.7∶44.5∶49.8,第三产业占比同比提高2.6个百分点。县域经济不断增强,地区生产总值、公共财政预算收入、社会消费品零售总额、固定资产投资占全市比重分别提高0.2、0.8、0.1、5.7个百分点。

(四)城乡人居环境提升。坚持规划指导,城市总体规划修改和历史文化名城保护规划经市人大审议通过并已报省,规划体系逐步完善。大力推进名城复兴工程,西城墙瓮城主体、南城墙景观主体、明堂公园主体、云冈五华洞窟檐保护工程完工。展览馆平移到位,博物馆正式开放。御东公共活动走廊铺装绿化完成年度任务,图书馆外立面装修完成。大力推进城镇提质工程,新建城市道路80条126公里。天镇—大同高速全线通车,灵丘—涞源高速已经完工,国道208一级公路改建基本完成。集中连片特困地区农村公路改造159公里。汽车客运东站开工。天镇、浑源等县城改道工程开工,大县城建设完成投资171.22亿元,6个省级、9个市级重点中心镇建设完成投资5.22亿元。大力推进生态建设工程,获得国家园林城市称号,广灵获得省级园林县城称号,大同县列入国家生态保护与建设示范区名单。建成区新增绿化面积119.33万平方米,完成营造林45.54万亩。国家环保模范城市创建规划通过评审,拆除燃煤锅炉156台,新增集中供热350万平方米、天然气居民用户5.7万户。二级以上天数300天,空气质量综合指数5.77,均列全省第一。国家卫生城市创建持续推进,餐厨垃圾处理厂投入运营,新建水冲式公厕13座、小型垃圾转运站15座。城乡清洁工程投资1.86亿元,问责65人次,常态化机制初步形成。被住建部、科技部确定为国家智慧城市试点。天眼工程投入使用。

(五)民生保障得到改善。大力推进收入倍增工程,公共财政预算用于民生支出达到80%,城镇新增就业5.6万人,失业人员再就业2.4万人,转移农村劳动力3万人,城镇登记失业率3%。提高最低工资标准和企业工资增长指导线。提高养老、医疗、工伤、失业、生育、低保等社会保障标准。提高市直机关、事业单位津补贴和绩效工资标准,并向低职级人员倾斜。社会救助体系进一步完善,为6992名失业人员和1.28万户困难家庭发放取暖补贴,为农户免费发放暖心煤74万吨,为2.76万户因病致困家庭发放救助金,为7639名贫困重度残疾人发放生活补贴。实施产业扶贫项目56个,完成投资22.5亿元,4.6万人脱贫。3.7万农村人口饮水条件得到改善。大力推进城乡安居工程,保障性住房新开工4.66万套,建成3.43万套。深入开展“安置年”活动,安置房竣工1.71万套,安置1.33万套、4万人,安置公产危房1045户。完成农村困难家庭危房改造4万户、农村住房抗震改建试点7740户、移民搬迁10630人。南郊口泉列入全省采煤沉陷区治理试点,开工搬迁治理项目14.4万平方米。大力推进百校兴教工程,市中小学社会实践教育基地、大同师范、大同铁一中建设工程基本完工,特教学校开工建设,改扩建村级幼儿园25所,新建标准化幼儿园24所、中小学操场55个。大力推进平安创建工程,安全生产经营性事故起数和死亡人数控制在考核目标内。归还政府到期贷款本息40亿元,协调解决拖欠农民工工资1.43亿元。

(六)发展环境不断优化。深入开展群众路线教育实践和学习讨论落实活动,严格落实八项规定,集中整治“四风”问题,开展专项整治30项,发布制度22项,压缩文件25%、精简会议20%,取消评比、达标、表彰项目168项,清退违规公务用车147辆。市本级“三公”经费支出下降27%。大力推进党风廉政建设和反腐败斗争,严肃查处了一批领导干部违纪违法问题和重点领域腐败案件。主动接受市人大依法监督和政协民主监督,办理人大代表建议165件、政协委员提案229件,办结率均为100%。完成3项地方性法规和政府规章起草论证工作。取消下放行政审批事项200项,强化政务大厅“两集中、两到位”,办结行政审批事项增长37.37%,办结率99.9%,办结建设工程各类审批事项增长1.7倍。12345政府服务热线运行良好,日均受理群众诉求750条,办结率98.9%。信访服务中心良好起步,公共资源交易大厅、廉政教育基地即将投用,人力资源市场加快建设。村委会、居委会换届顺利完成。舆情负面关注度下降46.24%,进京非访下降61.9%,社会大局稳定。

各位代表,这些成绩的取得,靠的是省委、省政府和市委的坚强领导,靠的是人大和政协的监督支持,靠的是广大干部群众的团结奋斗。在此,我代表市人民政府,向全市人民、驻同解放军、武警官兵、公安民警和驻同单位,向各民主党派、人民团体,向所有关心支持参与大同改革发展的各界朋友,表示衷心感谢和崇高敬意!

成绩来之不易,要倍加珍惜;问题不容忽视,更需高度警觉。一是经济下行压力比预想的更加严峻复杂,煤炭、电力、房地产三大主导产业量价齐跌,企业经营困难,社会投资意愿不强,固定资产投资增速全省倒数第一。二是县域经济规模不大,民营经济活力不强,城乡发展水平差距大,民生社会事业欠账多,扶贫攻坚难度大,信访维稳任务重。三是不确定风险因素明显增加,财政收支矛盾压力陡增,政府债务进入归还高峰,非法集资、恶意逃废债现象时有发生。四是政府依法行政能力滞后,政务环境不优,职能转变不到位,“四风”问题尚未根绝,不作为乱作为、懒政怠政、失职渎职行为依然存在。五是“两个责任”落实不力,发生多名市县主要领导干部腐败案件,严重损害了大同形象,党风廉政建设和反腐败斗争形势严峻、任务艰巨。对此,我们必须保持清醒头脑,增强忧患意识,树立必胜信心,采取有效措施,认真解决问题,推动改革发展,不负人民重托。

二、2015年工作安排

2015年是落实“四个全面”战略布局、推进“六大发展”总体目标的开局之年,是全面完成“十二五”规划的收官之年,做好今年工作意义重大。

当前,我市正处在资源型城市成熟期、工业化城镇化中后期、主导产业不振和政府债务沉重经济遇冷期、社会矛盾凸显和全面小康攻坚期“四期叠加”的特殊阶段,面临多方面的严峻挑战。但机遇与挑战同在,国家实施“一带一路”和京津冀协同发展战略,支持蒙晋冀毗邻地区(乌兰察布—大同—张家口)合作区建设,为我市带来深化区域合作的机遇。北京—张家口联合申办冬奥会将促进我市文化旅游产业发展。即将开工的大张高铁将使我市融入北京100分钟经济圈。国家稳增长系列政策将为我市促进经济转型起到重大拉动作用。党风廉政建设和反腐败斗争以及法治建设的深入推进,将为经济社会发展提供风清气正的环境。我们要直面挑战,抢抓机遇,奋发有为,努力工作。

今年政府工作的总体要求是:深入贯彻党的十八大、十八届三中、四中全会和习近平总书记系列重要讲话精神,按照“四个全面”战略布局,认真落实中央和全省经济工作会、全国全省“两会”和省委十届六次全会部署,坚持以提高经济发展质量和效益为中心,坚持稳中求进工作总基调,以六大发展为目标,以十大工程为抓手,以项目建设为引擎,科学认识、主动适应经济发展新常态,着力深化改革开放,着力推进三个突破,着力保障改善民生,着力强化风险防控,促进全市经济稳健发展、社会稳定和谐、人民安居乐业。

综合考虑各方面因素,2015年我市经济社会发展主要指标如下。

经济发展预期性指标:地区生产总值增长6%左右,全社会固定资产投资力争增长16%,社会消费品零售总额增长10%,公共财政预算收入预期下降3%、力争完成省下达增长3.4%的考核目标。

民生保障性指标:城市居民人均可支配收入增长7%,农村居民人均可支配收入增长8%以上;城镇新增就业岗位5.6万个,城镇登记失业率控制在4.2%以内,居民消费价格涨幅控制在3%左右。

安全环保约束性指标:万元地区生产总值能耗下降2.5%、二氧化碳排放量下降3%,万元工业增加值用水量下降5%;二氧化硫、氮氧化物、氨氮、烟尘、粉尘排放量、化学需氧量和安全生产指标完成省下达任务。

上述指标中,固定资产投资增速按照省不低于16%的要求,今年需完成1244亿元,我们必须全力以赴,千方百计,抓紧抓好。公共财政预算收入受多重不利因素影响,预测为负增长,省下达考核目标为增长3.4%,我们必须广开税源,应收尽收,力争完成。农民收入考虑到我市基数小、城乡差距大的实际,今年继续按高于全省一个百分点安排,增长8%以上,要想方设法,确保完成。

实现上述目标,必须重点做好以下工作:

(一)全面深化改革、扩大开放、加快创新,着力激发发展活力。改革增强动力,开放挖掘潜力,创新释放活力,三者互相促进,牵动全局,影响长远。要披坚执锐、敢破敢立,努力实现金融振兴、科技创新、民营经济三个突破。

深化金融体制改革,推进金融振兴取得突破。认真落实省市促进金融振兴的意见,不断优化金融生态,充分发挥驻同国有控股及股份制银行融资骨干作用,扩大融资规模,支持地方经济。推进大同银行市场化改革,吸纳高端人才,优化股权结构,尽快做大做强。加快农信社改制。鼓励民营资本设立民营银行和村镇银行,推动小额贷款公司健康发展。扶持新三板挂牌企业增资扩股、股权融通,争取早日转板上市;培育具备条件的中小微企业尽快完成股改,在新三板挂牌;加快推动小微企业在区域性股权交易市场(四板)挂牌。组建基金公司,设立旅游文化体育产业基金、战略新兴产业基金、中小微企业发展基金,探索建立创投基金参与城市投资机制。支持同煤财务公司进入全国银行间同业拆借市场。壮大市级政策性融资担保公司,加快发展现代保险服务业。用好十种化债方式,积极争取国家和省各类债券支持,化解存量债务,防范债务风险。

深化科技体制改革,推进科技创新取得突破。认真落实全省科技创新推进大会精神,深入实施创新驱动和低碳创新行动计划,构建以企业为主体、市场为导向、产学研用相结合的自主创新体系。加快创新企业孵化基地建设,新增企业技术中心4户、高新技术企业5户。实施重大科技专项,重点在重载货运机车、现代煤化工、光伏新能源、采煤新技术等领域取得进展,大力推广应用新技术、新材料、新设备、新产品,促进传统产业向安全、清洁、高效、低碳转型升级。积极引进国家“千人计划”“万人计划”领军人才,发挥晋投玄武岩院士工作站作用。用好基金杠杆,落实优惠政策,加快重点实验室、企业技术中心等创新平台建设。完善激励机制,让有贡献的科研人员合法致富。支持大同大学科技创新、成果转化,加强学科建设,发展短缺专业,为经济社会发展提供人才智力支撑。

深化商事制度改革,推进民营经济取得突破。进一步简化注册登记,逐步实现“三证合一”。积极搭建创业创新平台,大力培育各类创业主体,鼓励支持企业家“二次创业”,新增“小巨人”企业5户,培育“小升规”企业14户,创办小微企业1500户,新增市场主体22000户。坚持“非禁即入”原则,坚决拆除隐性壁垒,鼓励民间资本进入基础产业、社会事业等领域参与市场资源配置,放权、放手、放活,最大限度释放中小微企业主体活力,让创业创新遍地开花、“草根”经济枝繁叶茂。

深化其他重点改革。深化国资国企改革。整合古城资源、旅游资源、场馆资源,推进资产证券化,实现城市资源、资产变现、增值。坚持混合所有制改革方向,鼓励民间投资主体参与国有企业改制重组,加快竞争性国有企业股份制改造。两年内完成党政机关与所办企业脱钩。加快推广运用PPP模式,面向社会推介首批10大类项目包。激活社会资本,促进经济发展。深化三农改革。全面开展农村土地确权登记颁证,推进农村产权交易平台体系建设,总结阳高、天镇土地流转做法,引导农村土地有序流转,促进农业适度规模经营。加快推进左云、浑源等城乡建设用地增减挂钩、露天采矿用地、矿业存量土地整合利用试点,探索建立城乡统一建设用地市场。多渠道筹集资金开发土地1.6万亩。实施林光互补一体化改革,有效解决国家光伏示范基地建设用地问题。推进水利、国有林场、供销社综合改革。深化教育卫生体制改革。加快推进城乡义务教育均衡发展和医疗卫生优质资源均等化。继续深化纵向联盟、横向集团办学体制改革,实行校长、教师交流制度,鼓励教师扎根基层、奉献农村。稳步推进招生制度改革,加强学校班子任期目标考核。进一步加强医疗联合体建设。全面推进县级公立医院综合改革。启动浑源、大同两县县乡医师一体化试点。推进优质医卫资源下沉,鼓励和规范医师多点执业,加强村医队伍建设和卫生人才培养,实行分级诊疗制度,方便群众就近看病。同时,积极推进区划调整,启动不动产登记,加快价格、文化、住房公积金制度改革。

实施全面开放战略。持续推动航空口岸正式开放,建设无水港,争取陆路口岸开放,全力支持大秦公司发展,增开国际、国内客运航线,强化全国综合交通枢纽城市地位,打造对外开放大通道。用好国家级经济技术开发区金字招牌,设立左云省级经济技术开发区,提升各级各类园区功能,构建承接项目大平台。不断创新招商方式,充分发挥同煤、国电、大唐、国药、京能等驻同省企、央企、京企优势,精准招商、以企招商、以商招商,落实各项政策措施,促进招商引资大提质。完成乌大张区域合作规划编制,办好第二届乌大张区域合作年会,主动融入京津冀,积极对接“一带一路”,推动区域合作大发展。

(二)以百企强市、百园立农、名城复兴工程为抓手,着力推动转型发展。按照产业基地化、基地园区化、园区项目化思路,加大特色优势产业基地建设力度,加快构建“1+13+1”产业体系,推动经济稳增快转。

全力推动项目建设。产业项目不足是长期制约我市发展的最大因素,投资增速低缓是我市当前经济增长的主要问题。要围绕十大领域,坚持“六位一体”,扎实开展项目提质增效年活动,全力推进固定资产投资增长。建设一批大项目,投资50亿元的同煤塔山二期低热值煤发电项目主体和设备安装基本完成,投资33亿元的同煤阳高低热值煤发电项目主体完工50%,投资53亿元的京能左云低热值煤发电项目实现建筑封顶,投资61亿元的采煤沉陷区治理项目完成投资10亿元。开工一批大项目,投资182亿元的大张高铁控制性工程8月开工,投资258亿元的煤制气项目加快完善环评、年内开工,投资52亿元的无水港进口肉类口岸查验场8月开工,投资32.7亿元的同煤浑源热电联产和投资30亿元的同煤大唐热电三期项目争取尽快取得路条、年内开工,投资80亿元的国电湖东电厂力争取得路条、年内开工。投资10亿元的信息产业园8月开工。投资15亿元的引黄入左项目力争9月开工。投资100亿元的同煤烯烃项目环评获批、力争早日开工。投资289亿元的300万千瓦光伏示范基地8月开工,利用3年时间,建成国家光伏新技术示范地、领跑技术实践地、先进技术聚集地。谋划一批大项目,投资50亿元的浑源抽水蓄能调峰电站9月完成预可研、力争明年开工,投资35亿元的大唐御东电厂年内取得路条。千方百计推进城市轻轨、雁同灌区、大同—原平客专、乌兰察布—大同客专、广灵—山阴地方铁路等项目前期,力争早核准、早开工。大张高铁生命线项目、煤制气转型标志性项目、低热值煤发电产业发动机项目、新能源绿色革命项目、无水港开放战略项目,历经艰苦努力,实现同年开工,迈出转型发展坚实步伐。

做好煤炭这篇大文章。落实好省市深化煤炭管理体制改革意见,推动煤炭消费、供给、管理、科技革命,加快煤炭产业“六型”转变,建设国家新型综合能源基地。同煤东周窑、金庄2座千万吨矿井加快建设步伐,高山、韩家洼等5座矿井通过竣工验收,口泉煤业、梅花沟2座矿井基本建成进入联合试运转,新增产能510万吨。加快热电联产、低热值煤发电、超超临界参数机组、特高压外送通道项目建设,打造千万千瓦级火电基地。同煤60万吨甲醇、10万吨活性炭项目达产达效,同车10万吨活性炭项目一期试生产,争取开工二期;培育壮大华青、光华等一批活性炭潜力企业,打造晋北煤化工基地。进一步抓好塔山循环经济园区建设,推动山西聚力节能环保产业园早日落地,大力发展粉煤灰新型材料、高岭土、脱硫石膏等产业,加大秸秆、城市垃圾等废弃资源综合利用,打造资源综合循环利用和节能环保产业基地。

推动非煤产业发展。坚持园区化、基地化发展路径,加快基础设施建设,优化配置要素,促进产业发展。重点抓好以威奇达为龙头的百亿元抗生素生产,以普德、同达、振东泰盛为骨干的现代医药生产,打造特色医药产业基地。支持北方通用动力、大齿、陕汽、北宇等企业加快发展,帮助同车争取30吨货运电力机车批量生产订单,扶持同煤煤机、同华矿机、唯实机电、北方煤机等项目建设,打造现代装备制造产业基地。稳定山纳氯丁橡胶产量,扩大晋投玄武岩生产,建成新成新材料公司高速列车受电弓滑板项目,积极鼓励研发石墨烯新材料、珍珠岩保温板材,打造新材料产业基地。推动大同县轻型飞机制造产业园、灵丘通用航空产业园、阳高通用机场建设,培育轻型飞机制造销售、航空展览旅游、航空人才培训产业集群,打造通用航空产业基地。叫响夏都品牌,依托5个三甲医院优质资源,积极发展养老服务相关产业,大力扶持普德食品公司等养生企业,打造智慧生态养老基地。积极发展互联网+商贸物流,出台商业网点规划,增添商业新型业态,规范物流市场管理,支持新发地、东信城物流园区发挥更大作用,壮大万昌、金洋等物流园区,加快亿丰世贸中心项目建设,支持华林、银星金店等健康发展,扶持啤斯、青松食品做大做强,打造商贸物流产业基地。加快培育消费增长点,鼓励大众消费,壮大信息消费,提升旅游消费,稳定住房消费,扩大文化消费,遏制消费持续下滑局面。

加快发展现代农业。加大农田建设保护力度,建设高标准农田20万亩。新增设施农业3万亩。推广旱作节水农业20万亩、地膜覆盖200万亩,发展膜下滴灌1.4万亩。推进马铃薯主粮化,抓好粮改饲与种养结合模式试点。加快伊磊牧业、中地奶牛、政通10万只肉羊等亿元园区建设。继续推进养羊“五个一”计划,新建扩建规模化养殖小区200个,实现奶牛存栏10万头、猪饲养量290万头、羊饲养量680万只。大力扶持农业产业化“513”龙头企业,支持菲尼克斯特色农业园区、北方四季牧场马身猪基地发展壮大。加快构建农产品电子商务平台,推行农企、农超、农校对接等产销衔接模式,支持天镇特色农产品北京东城区直营店擦亮品牌、扩大规模,打造京津特色米袋子和绿色菜篮子供应基地。加大农产品“三品一标”认证。鼓励县区积极申报“中国杂粮之乡”。

做大做强文化旅游产业。依托北京,借力冬奥,打响“五都”牌,建设国际旅游目的地。在明堂遗址公园建设平城丝绸之路博物馆,在云冈景区设立北魏佛教文化博物馆,在代王府建设古建筑艺术博物馆。支持西京博物馆建设,鼓励社会力量建馆办馆,促进文博事业蓬勃发展。加快西城墙合拢,完善北魏398文化创意园、古城文化艺术影视基地、御东五大文化场馆、火山群地质公园。方特欢乐世界正式开园迎客。办好第25届全国图书交易博览会(大同分会场)、云冈文化旅游节等活动。办好“影像的力量”中国(大同)国际摄影文化展。大力发展红色经典、美丽乡村、特色工业、生态观光、休闲度假、冰雪体验、自驾骑行等旅游业态。做专做特酒店、餐饮服务业。启动全国旅游标准化示范城市创建。加大扶持力度,整合旅游资源,发展壮大旅行社,联手知名旅游网站,推介旅游产品和精品线路,提升大同旅游品牌。

推动县域经济发展。县域经济不强是制约我市经济发展的最大短板。各县要立足资源禀赋、区位条件和产业基础,形成1~2个主导产业。左云县要加快千万吨级煤矿、煤制气等项目建设进度,打造大同工业经济增量的半壁江山。大同县要服务园区发展,做大黄花产业,优化生态环境,发展城郊型经济。浑源县要一手抓煤炭产业,一手抓旅游等现代服务业。灵丘县要提升冶金、新材料产业,发展通用航空、有机农业。阳高县、天镇县、广灵县要发展特色农业、文化旅游和养老养生产业。实行“飞地经济”,实现项目按规划定位、跨县域承接,培育我市新的增长极。

(三)以城镇提质、城乡安居、生态建设、城乡清洁工程为抓手,着力改善城乡人居环境。统筹城乡是新型城镇化的本质要求,要努力实现建设重点由重视城市向统筹城乡转变,城建方式由外延式向内涵式转变,城市发展由建设为主向建管并重转变,持续推进四城联创。

加强城乡基础设施建设。城市总体规划修改、历史文化名城保护规划争取早日获批,历史文化街区保护规划、文瀛湖环湖区域概念规划等专项规划适时出台,实现规划指导下的科学发展。深入实施城乡人居环境改善工程,完成投资193亿元。城市道路新建续建80公里,泉新路建成通车,北环桥主体完工,开源桥主体完成70%。大剧院、美术馆、体育中心加快建设。灵丘—涞源高速建成通车,京新高速山西段(天镇)主体完工。实施国省道改建改线改造8条158公里。新开公交线路8条,更新出租车300辆。汽车客运东站主体完工。完成农村公路改建125公里、县乡公路改造142公里。新建老年人日间照料中心75个,完成广播电视卫星户户通5万户。办好学校、医院,增强县城辐射功能,全力推进大县城建设。推进县级图书馆、文化馆、农家书屋和乡镇文化站等基础设施建设。抓好农村饮水安全和农网升级改造。通过多种方式落实社区办公用房。

切实改善城乡居住条件。加大棚户区和城中村改造力度,新开工保障房7.92万套,基本建成3.54万套。实现市区房屋征收住户应安置尽安置(1万户),改造老旧小区100万平方米、1.2万户。农村住房抗震改建9500户、困难家庭危房改造1.57万户、地质灾害治理搬迁256户。抓好南郊、新荣、左云、浑源4区县26个村的采煤沉陷区治理。激活住房公积金存量资金28亿元。加强房地产市场监管,规范物业管理招投标,着力解决产权办证难题,维护购房者合法权益,促进房地产业健康发展。

持续创建国家环保模范城市。推进同煤“两区”生活污水处理厂建设、同煤生活污水处理分厂提标改造,改善甘河、口泉河跨界断面水质。加强御东污水厂市场化运营监管。严格实施大气污染防治2015行动计划,以控煤、治污、管车、降尘为重点,加强源头防控。大唐云冈、大唐塔山、同煤同达等电厂完成超低排放改造。加大机动车尾气污染防治,加快淘汰黄标车、老旧车。深入开展整治违法排污专项行动,严厉查处禁燃区燃煤锅炉、古城内原煤散烧及工地扬尘、垃圾秸杆焚烧等各类环境违法行为。全面落实创模验收关键指标。完成南城墙景观绿化、御东公共活动走廊建设和儿童公园改造一期工程,建成区新增绿化面积150万平方米。大泉山展览馆建成开馆。完成村庄绿化157个,新增营造林32万亩。完成水保治理14万亩。抓好采煤沉陷区生态修复。让蓝天、白云、青山、绿水成为常态。

持续创建国家卫生城市。以城区、矿区、南郊区和开发区为主体,以旱厕改造和集贸市场规范建设为重点,扎实推进基础设施建设、环卫专项整治和创卫宣传教育,力争完成申报、通过省级验收。餐厨垃圾厂日处理能力达到60吨,富乔生活垃圾焚烧发电厂增加日处理能力500吨。城市新增集中供热面积100万平方米,供热计量收费面积达到650万平方米。推动城市污泥、粪便处置项目。建成新荣区、大同县、天镇县垃圾无害化处理场和农村垃圾处置点300座,持续改善农村人居环境。

持续创建国家新能源示范城市。开工建设投资100亿元装机100万千瓦国家光伏示范基地项目;完成投资35亿元的8万千瓦光伏扶贫项目、6万千瓦分布式项目、6万千瓦地面常规项目、17万千瓦结转项目,新增装机37万千瓦;完成投资50亿元的风电项目,新增装机60万千瓦。年内开工新能源装机197万千瓦,实现总量翻番。新增二星级绿色建筑10万平方米,完成既有居住建筑供热计量与节能改造100万平方米。创建御东新区国家绿色生态示范区。

持续创建国家智慧城市。以“互联网+”为重点,整体规划,分步推进,实施智慧城管、智慧社区、智慧教育、智慧医疗、智慧旅游、智慧物流、智慧照明、智慧养老项目。管好用好天眼工程。完善中小学校园网配套设施,完成400所乡镇以下学校互联网接入工程。建成“扁鹊飞救”智慧医疗项目。加快建设城市公共信息平台和公共基础数据库一期工程,办好电子商务园区,新增电商企业150家。

(四)以百校兴教、收入倍增、平安创建工程为抓手,着力保障改善民生。更加注重社会公平公正,认真解决人民群众切身利益问题,使发展更具普惠性,让群众更有幸福感。

推动各类教育协调发展。均衡发展义务教育。加快推进县域义务教育均衡达标,左云县通过教育部达标认定。铁一中等4所中小学建成投用,完成中小学社会实践教育基地以及二十中等5所学校标准化体育场建设。加快大同六中分校、同煤一中初中部等项目前期,启动二十一中新建项目。严格规范中小学招生,严肃整治教育“三乱”行为。大力发展职业教育。完成大同师范、艺校工程建设,办好阳高、浑源、左云3个区域职教中心,鼓励发展轨道交通、物流商贸、航空服务、导游、家政等专业,卫校养老护理专业今年秋季招生。充分发挥大同煤炭职业技术学院、同煤技师学院等职业院校服务地方经济的独特作用。加快发展学前教育,新改扩建标准化幼儿园16所、村级幼儿园24所。落实土地等各项政策,鼓励发展民办教育。

提升医疗卫生服务水平。支持市六医院升级三甲,鼓励中医院对外协作,加快建设市急救中心和中心血站,启动建设全科医师临床培训基地。继续加强医疗专科建设。组织实施二级医院对口支援乡镇卫生院项目,开展公立医院帮扶社区卫生服务机构试点。进一步加大城乡医疗保障力度。持续关注因病致贫返贫困难人群,特殊病种大额门诊补偿增加到35种,重大疾病保障增加到24种。进一步整顿规范医疗市场,做好重大传染病及疫情防控工作。

努力促进城乡居民增收。越是经济下行,越要关注民生,特别是低收入、零就业家庭和农村贫困人口。坚持就业优先,发展吸纳就业能力强的产业,发挥中小微企业带动就业的作用,推进创业带动就业,促进高校毕业生、零就业困难家庭等重点群体就业。推行企业职工工资集体协商制度,落实最低工资标准。提高机关事业单位职工工资水平,增加机关事业单位离退休人员离退休费,落实县以下基层公务员职务职级并行制度。扶持发展特色高效农业,提高农民经营性收入;不折不扣执行强农惠农政策,提高农民转移性收入;推动土地要素资本产权制度改革,提高农民财产性收入;做好农村劳动力转移工作,支持发展天镇家政、大同技工、广灵剪纸、浑源搓澡、阳高刺绣等劳务品牌,扩大向京津地区劳务输出,努力增加农民务工性收入。加大拖欠农民工工资治理力度。

完善社会保障体系。稳妥推进机关事业单位养老保险制度改革。按政策及时提高各项保险待遇水平。企业退休人员基本养老金标准提高10%,城乡居民基础养老金提高15元,城镇居民医保人均财政补助标准提高60元,城、乡低保标准分别提高7%、11%,农村五保户集中、分散供养标准每人每年分别提高200元、100元。完善城镇医保省内异地就医管理制度。完成城镇医保大病招投标工作。高度关注农村留守儿童、老人、妇女工作。积极发展社会福利和慈善事业。

全力推进扶贫攻坚。贫困人口多、贫困程度深、扶贫任务重是我市的基本市情。抓好同煤等38个重点企业产业扶贫项目,完成天镇、浑源光伏产业扶贫建设任务。推进浑源县荆庄村等4个省级乡村旅游扶贫项目。大力推广电商扶贫,实施好金融扶贫。培训新型职业农民3700人、“千村万人”计划就业2000人。推进整体易地移民搬迁10697人。考取二本B类以上贫困户学生一次性资助5000元。按照精准扶贫的要求,充分发挥各级党委政府的主体责任、驻村扶贫工作队的协调作用,确保帮扶到村、到户、到人。同时,要充分调动贫困人口主体的积极性。

持续抓好安全生产。要敬畏生命、敬畏责任、敬畏制度,牢固树立安全第一理念,严格落实“六位一体”安全生产责任。扎实做好煤矿、非煤矿山、道路交通等十大重点行业安全生产工作。继续保持打击私挖滥采、非法生产、违法建设三个高压态势,深入开展安全生产大检查、隐患排查整治、危旧楼房普查和“专家会诊”专项行动。不断加强食品药品安全监管,建好市县乡三级执法监管队伍,建立质量安全可追溯制度,保障群众“舌尖上的安全”。

不断加强社会治理。我市处在上访高发、“上街”多发、网上负面舆情频发的社会矛盾凸显期。要畅通信访渠道,规范信访秩序,引导依法逐级上访。严格执行领导包案化解信访案件制度,认真开展专项治理活动,及时防范和妥善处理信访突出问题及群体性事件。依法打击网上恶意炒作、发布虚假信息行为,依法打击恶意欠薪、逃废债、非法传销、非法集资行为,依法打击无理缠访闹访、幕后组织操纵群访行为,依法打击各类阻工行为,创优发展环境,建设平安大同。

支持国防和军队建设,推进军民融合深度发展,做好优抚安置和人防工作,争创全国双拥模范城八连冠。落实全民健身计划,构建全民健身服务体系,提高人民健康水平。发展妇女儿童、老龄、红十字会等事业。加强防震减灾、气象、史志、档案、科普等工作。做好民族宗教、外事、侨务、港澳、对台等工作。

三、全面加强政府建设

扎实开展“三严三实”专题教育,认真履行“两个责任”,全面推进“六权治本”,努力建设法治政府、廉洁政府、创新政府、服务政府。

(一)建设法治政府。坚持把依法合规作为行政的根本准则。全面依法履行职能。主动接受人大及其常委会监督和政协民主监督,认真办理人大代表议案、建议和政协委员提案。制定实施政府建设三年规划,推动政府建设系统化、规范化、法治化。抓住五个重点,深化政府机构改革,推动市直事业单位分类改革。抓住五个环节,深化行政体制改革,进一步简政放权,主动承接国家和省取消下放行政审批事项。年内公布市级权力清单、责任清单,明年一季度完成县级“两个清单”。全面完善依法决策机制。严格按照五个程序研究决定重大事项、重大项目、重大决策。认真总结“十二五”规划完成情况,科学编制“十三五”规划。建设政府智库,完善政府决策咨询制度。健全重大行政决策终身责任追究制度及责任倒查机制。全面规范行政执法。加强政府立法工作,建立健全法律顾问制、公职律师制,严格落实规范性文件备案审查制。全面落实行政执法责任制,探索综合执法改革,完善市县两级行政执法管理,健全行政裁量权基准制度,高度重视、认真做好行政复议、行政应诉工作。全面推进政务公开。严格实行政府信息公开,严管公共资金,把政府收支全部纳入预算管理,推进预决算公开和“三公”经费公开。完成公车改革,规范办公用房管理。全面推进依法治市。深入开展法治城市、法治县区、法治示范村创建活动,落实一村(社区)一法律顾问制度。推动市、县法律援助便民大厅和基层法律援助站点全覆盖。各级政府要依法行政、按章办事,所有政府工作人员要秉公执法、遵纪守法,社会各界要办事依法、遇事找法,加快建设法治大同。

(二)建设廉洁政府。坚持把清正廉洁作为从政的根本要求。强化纪律。把纪律和规矩挺在前面,严守政治纪律和政治规矩,保持政治定力。严格执行组织、财经、工作、生活等各项纪律。强化责任。从严落实政府党组的主体责任、党组书记的第一责任和政府班子成员的“一岗双责”,把党风廉政建设作为应尽之责,管好班子,带好队伍。持之以恒纠正“四风”,严肃查处懒政、怠政和腐败问题,维护群众切身利益。强化监督。加强行政监察和审计监督,重视社会和舆论监督。抓住八个关键环节,紧盯六个重点领域,强化部门和岗位监督。使讲规矩、守纪律成为每一位政府工作人员的行为准则。

(三)建设创新政府。坚持把改革创新作为理政的有效举措。强化行政基础设施建设。充分运用好政务大厅、公共资源交易平台、人力资源市场、12345政府服务热线、廉政教育基地、信访服务中心六个平台,推进场所、设施、机构、职责、人员到位,加快事项办结,加大绩效考核,加强监督问责,提高服务质量。创新管理方式。推行“互联网+政务”,加快政府OA办公系统二期建设,整合市县政府网站资源,建成大同政务云。推进“五规合一”,建立多部门联动审批机制,解决项目审批慢、落地难的问题。创新与公众沟通方式,积极回应社会关切。改革公共服务提供方式,拓宽教育、医卫、社保、养老、文体、助残等政府购买服务领域。

(四)建设服务政府。坚持把“三严三实”作为治政的基本遵循。加强作风建设。扎实开展专题教育,不断加强内外兼修,始终坚持抓常抓长,自觉做到忠诚干净担当,实实在在谋事创业做人。加强公务员队伍建设,提高依法行政水平。改进调查研究方法,增强服务群众本领,切实把好事实事真正办到群众心窝里。狠抓工作落实。坚持对标一流定目标、一事一表做计划、按月调度抓落实、活力曲线抓考核,强化横向到边、纵向到底、责任到人的项目化管理,全力推动十大工程。践行“五个坚持”,突出“五个必须”,认真督办督查,保障工作落实。

各位代表,信心凝聚力量,实干成就事业。让我们紧密团结在以习近平同志为总书记的党中央周围,在省委、省政府和市委的坚强领导下,万众一心、攻坚克难,脚踏实地、埋头苦干,为实现弊革风清、富民强市目标而努力奋斗!
